# Diagnostic Performance of Serum Human Epididymis Protein 4 (HE4) for Prediction of Malignancy in Ovarian Masses 

**DOI:** 10.31557/APJCP.2019.20.4.1103

**Published:** 2019

**Authors:** Rupali Dewan, Abhinav Dewan, Meera Jindal, Mausumi Bhardawaj

**Affiliations:** 1 *Department of Obstetrics and Gynaecology, Vardhman Mahavir Medical College and Safdarjung Hospital, Ansari Nagar, *; 2 *Department of Radiation Oncology, Rajiv Gandhi Cancer Institute and Research Centre, Sector-5, Rohini, New Delhi,*; 3 *Department of Research, National Institute of Cancer Prevention and Research, Noida, Uttar Pradesh India. *

**Keywords:** Ovarian masses, malignancy, human epididymis secretory protein E4, CA-125

## Abstract

**Background:**

Early diagnosis of ovarian cancer is essential for long term disease control and mortality reduction. This has been achieved using tumor markers like cancer antigen 125 (CA-125) which is elevated in malignant as well as non-malignant conditions. This dilemma led to efforts towards development of newer markers like serum human epididymis secretory protein E4 (HE4). Present study aimed to evaluate role of HE4 in diagnosing ovarian cancers and comparing it with CA-125.

**Methods:**

Serum samples from 67 patients with ovarian cancer, 42 with benign ovarian masses and 26 healthy controls were collected preoperatively and tested for serum HE4 levels and CA-125 levels. Diagnostic performance of both tumor markers (HE4/CA-125) to diagnose malignancy in ovarian masses was calculated and compared to each other.

**Results:**

Mean CA-125 and HE4 levels were significantly higher in patients with ovarian cancer than in those with benign disease (p<0.001) or healthy controls (p< 0.001). Serum HE4 levels significantly increased in epithelial ovarian cancers when compared to non-epithelial ovarian cancers (p<0.01). Using benign control as comparison, receiver operating characteristic curve (ROC) was generated to predict a cut-off value for diagnosing malignancy for serum HE4 and CA-125. Compared to CA-125, HE4 had a similar sensitivity (83.6% vs. 85.10%) and higher specificity (100% vs. 90.48%); combination of serum HE4 and CA-125 improved the sensitivity to detect ovarian cancer to 92.54%. Sensitivity of HE4 to detect early stage ovarian cancer was superior to CA-125 (92.61% vs. 63.41%).

**Conclusion:**

Serum HE4, a novel tumor marker, discriminated epithelial ovarian cancer from benign ovarian masses. HE4 levels were related to the stage and histological types with the lowest levels in mucinous epithelial ovarian cancer and non-epithelial malignancy. Measuring serum HE4 levels alongwith CA-125 may provide higher accuracy for detecting epithelial ovarian cancer particularly in the early stages.

## Introduction

The primary goal of diagnosis of ovarian adnexal mass is to determine whether it is benign or malignant. It is estimated that 5-10% of women undergo surgical procedure for suspected ovarian neoplasms and 13-21% of these women are found to have an ovarian malignancy (Curtin, 1994). Accurate pre-operative discrimination between benign and malignant adnexal masses would help to optimize surgical management of women with pelvic tumors (Junor at al., 1994). Appropriate first line surgery has a great influence on the prognosis of women with ovarian cancer. The initial laparotomy is not only important for the accurate determination of the extent of disease, but also presents the best opportunity for maximum debulking (Raja et al., 2012). 

A non-invasive means of discriminating between malignant and benign pelvic masses can be achieved by various methods including tumor markers, grey scale ultrasound features and doppler ultrasound. Serum tumor markers have been investigated for their potential role in distinguishing benign from malignant masses. The most widely used marker is serum cancer antigen-125 (CA-125), which is raised in about 80% of the epithelial ovarian cancers. However, serum CA-125 levels alone are relatively non-specific and have therefore always required interpretation in conjunction with clinical and ultrasonographic (USG) findings. The use of CA-125 for detection of ovarian cancer in pre-menopausal women is associated with a low sensitivity and specificity; but has found more useful application in post-menopausal cases (Moss et al., 2005). 

The diagnostic sensitivity of CA-125 in ovarian cancer is related to the tumor stage, with abnormal CA-125 serum concentration seen in approximately 50% of patients with stage I disease and 80–90% of patients with stages III–IV disease (Nustad et al., 1996; Li et al., 2009). High CA-125 concentration may be found in malignancies of different origin, including non-ovarian gynecologic cancers and benign gynecologic conditions such as myomas and endometriosis (Hussain et al., 2004; Bast et al., 1998; Dilek et al., 2005; Meden and Meibodi, 1998; Ismail et al., 1994; Cheng et al., 2002; Kitawaki et al., 2005; Abrao et al., 1999). CA-125 concentration may fluctuate throughout the menstrual cycle and pregnancy (Spitzer et al., 1998; Bon et al., 1999).

Recent studies indicate that human epididymis protein (HE4) was encoded by gene WFDC2 and was overexpressed in ovarian cancer (Moore et al., 2009; Galgano et al., 2006; Moore et al., 2008., Kirchhoff et al., 1991; Kirchhoff et al., 1998). Results of previous studies have shown that HE4 has similar sensitivity, but higher specificity than CA-125 for the diagnosis of ovarian cancer (Wang et al., 2014).

In this study, we aimed to evaluate the diagnostic ability of serum HE4 in detection of malignancy in women presenting with ovarian adnexal mass and compare diagnostic utility of HE4 with that of CA-125 in ovarian cancer. In addition, the present study aimed at finding out whether the diagnostic accuracy to detect ovarian malignancy can be improved by incorporating serum HE4 to existing tumor marker (CA-125). 

## Materials and Methods

We conducted a prospective observational study in the department of obstetrics and gynaecology, Vardhman mahavir medical college and safdarjung hospital, New Delhi, India, in collaboration with the Institute of cytology and preventive oncology, Noida, Uttar Pradesh, India.

Study group comprised of 109 patients with adnexal masses of ovarian origin, diagnosed on clinical examination and ultrasonography and who were then subjected to surgery. Twenty six age matched healthy subjects were included as controls and serum levels of tumor markers were measured. Inclusion criteria included 1) age between 18-70 years, 2) patients with adnexal mass of ovarian origin on pelvic imaging and 3) patients scheduled for surgical exploration of adnexal mass. Those women in whom no surgical removal of ovarian mass was done, women with delayed surgical intervention >30 days after ultrasonographic examination, women with history of bilateral oophorectomy, pregnant women, women with history of abdominal Koch’s and previous malignancy and those who refused consent were excluded from the study. Study was approved by ethics committee of Vardhman mahavir medical college and Safdarjung hospital, Delhi. 

*Methodology*

Women fulfilling the specified inclusion and exclusion criteria were enrolled in this prospective observational study. Detailed history and physical examination was done. Clinical and histopathological parameters were recorded for each woman.

About 10 ml of peripheral venous blood was obtained preoperatively from all women and examined for serum CA-125 and HE4 levels. Samples were collected in a vacutainer, clotted for 60–90 minutes and centrifuged for 10 minutes. Serum fractions were aliquoted and stored at −80°C. Samples were later assessed at Institute of cytology and preventive oncology, Noida by chemiluminescent microparticle immunoassays specific for HE4 and CA-125. A standard cut-off value for HE4 of 70pmol/L was considered as suggested by Moore et al., (2008). Suggested cut-off for CA-125 to detect ovarian malignancy was 35U/ml (Bast et al., 1983). Area under curve (AUC) was calculated to determine our own cut-off values. 

Patients were staged according to International federation of gynaecology and obstetrics (FIGO) surgical staging (Prat and FIGO Committee on Gynecologic Oncology, 2014). Postoperatively, histopathological examination was performed and reported as per the WHO histopathological classification of ovarian cancer for all women (Kaku et al., 2003). Mean value of tumor markers was calculated in the ovarian cancer and control group. Data was further analysed according to stage, histopathology, myometrial invasion and lymph node involvement. Ability of diagnostic models to detect malignancy was tested prospectively and correlated with the histopathological factors.

Statistical analysis was performed by the SPSS program for Windows, version 23.0. Continuous variables were reported as mean ± SD, and categorical variables as absolute numbers and percentage. Normally distributed continuous variables were compared using the paired t-test, whereas Mann-Whitney U test was used for those variables that were not normally distributed. Categorical variables were analysed using either the Chi-square test or Fisher’s exact test. Diagnostic performance (Sensitivity, specificity and predictive values) of serum HE4 and CA-125 was calculated and compared to each other. A p-value<0.05 was considered statistically significant.

## Results

There were 67 malignant ovarian carcinoma patients, including 54 with ovarian serous adenocarcinoma, 8 with mucinous adenocarcinoma and 5 cases of non- epithelial ovarian malignancy. The surgical staging according to FIGO staging criteria was 41 cases of stages I/II and 26 cases of stages III/IV. There were also 42 patients with benign ovarian tumors, including 16 with ovarian serous adenoma, 22 with mucinous adenoma, 2 with benign ovarian teratoma and 2 with endometriotic cysts. Mean age was statistically higher in the malignant ovarian cases in comparison to the benign counterpart. 

*Comparison of Serum HE4 levels of each group*

Serum HE4 was 96.76±38.96pmol/l in the ovarian cancer patients, 49.33±10pmol/l in the benign ovarian tumors and 47.97±9.43pmol/l in the healthy individuals. Serum concentration of HE4 levels of ovarian cancer patients was significantly higher than benign and healthy control group (p<0.001). Difference between the HE4 serum levels of benign ovarian tumor and healthy cohort was not statistically significant (p>0.05). The results are shown in Table 1.

*Association between serum HE4 levels, clinical staging and pathological types of ovarian cancer*

Serum HE4 levels were significantly higher in the stage III/IV when compared to stage I/II and the difference was statistically significant (p<0.001). The levels were highest in the serous adenocarcinoma and the difference was statistically significant (p<0.001) when compared with other histological types. 

**Figure 1:**
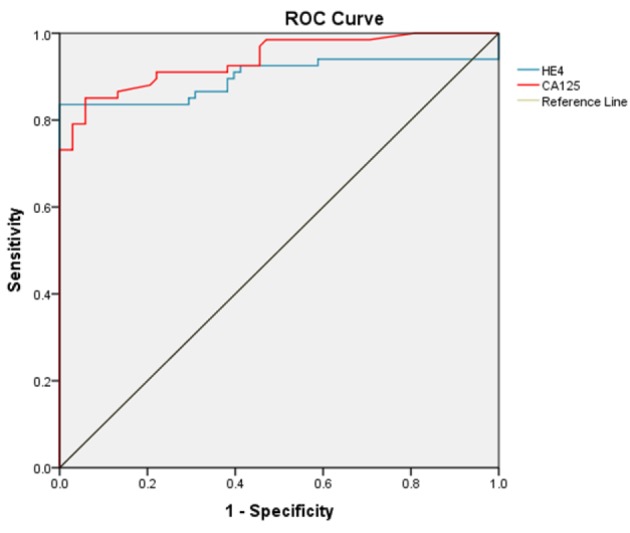
Receiver Curve Analysis (ROC Curve) to Predict Diagnostic Accuracy of Serum HE4 and CA-125 in Diagnosing Ovarian Cancer

*Diagnostic value of serum HE4 for ovarian cancer (* Table 3 *,* Figure 1 *)*

ROC curve analysis: To predict ovarian cancer, an ROC curve was used to determine a cut-off value of serum HE4 and serum CA-125. Analysis revealed a cut-off value of 69.8pmol/l for HE4 (AUC 0.899, 95% confidence interval 0.836-0.962, sensitivity 83.6%, specificity 100%) and 33.55 U/ml for CA-125 (AUC 0.939, 95% confidence interval 0.900-0.979, sensitivity 85.10%, specificity 90.48%) to predict malignancy. 

*Comparison of Diagnostic Values of Serum HE4 and CA-125 for ovarian cancer (* Table 3 *,* 4 *)*

In ovarian cancer group, HE4 levels above the cut-off were detected in 56/67 patients when compared to CA-125 levels that were detected above the cut-off value in 57/67 patients. HE4 levels above cut-off were never detected in benign and control group, whereas CA-125 levels above cut-off were present in 2 cases in the benign group. The overall sensitivity of CA-125 in detecting ovarian cancer patients was higher. The specificity and positive predictive value of HE4 is 100%, whereas CA-125 has a lower specificity (90.48%) with a positive predictive value of 93.44%. 

The specificity and sensitivity of HE4 for early stage ovarian cancer was higher when compared to the CA-125. Sensitivity of CA-125 was more than HE4 for higher stage ovarian cancer (96.41% vs 92.59%). HE4 was able to diagnose 38/41 early stage cancers as compared to 26/41 cases as diagnosed by CA-125. 

HE4 was not able to diagnose 1 case of serous cystadenocarcinoma, 5/8 cases of mucinous cyst adenocarcinoma and all the cases of non-epithelial ovarian malignancies. CA-125 was unable to diagnose 6 cases of serous cystadenocarcinoma, 1 case of mucinous and 3 cases of non-epithelial cancers. 

On combining CA-125 and HE4, 62/67 cases of ovarian cancers were detected and the sensitivity increased to 92.54%. 

## Discussion

The present study showed that the concentration of HE4 in ovarian cancer patients was significantly higher than that in benign ovarian tumor and normal control group (p<0.001), and no statistically significant differences was observed (p>0.05) between the benign ovarian tumors and normal control group. The results of the present study were consistent with those of Moore et al., (2009) and Köbel et al., (2008) who observed that the serum levels of HE4 were significantly increased in the epithelial ovarian cancer patients. The mechanism of HE4 overexpression in ovarian cancer is not clear. However, the results of Berry et al., (2004) showed that the chromosomal region where HE4 was located was frequently amplified in the breast and ovarian cancer patients. HE4 was not expressed in the normal ovaries or fallopian tubes. Wang et al., (1999) studied the expression of HE4 in various ovarian tissues and observed that HE4 was highly expressed in the cancer tissue, but not in the normal ovarian tissue. Thus, the level of serum HE4 may be used as a marker for the diagnosis of ovarian cancer. 

The ROC curve analysis on healthy controls and patients with ovarian cancers revealed that HE4 had a significantly higher AUC when compared with CA-125 (0.89 vs 0.93). We aimed to find the cut-off points for HE4 and CA-125. The cut-off values corresponding to the highest accuracy (minimal false-negative and false-positive results) for all patients were 33.5 U/ml for CA-125 and 69.84 pmol/l for HE4. At this cut-off of 69.84 pmol/l (Close to 70 pmol/l as suggested by Moore et al., (2008)), sensitivity and specificity of HE4 to detect ovarian cancer was 83.6% and 100%, respectively. The sensitivity of HE4 in detection of the ovarian cancer was comparable to CA-125, but specificity was higher than CA-125 (Table 4). Thus, HE4 was considered a promising ovarian cancer marker. Similar finding was reported in a meta-analysis by Yu et al., (2012). This was therefore comparable to our ideal cut-off point of 70 pmol/l, and was thus a reasonable cut-off point for HE4.

**Table 1:** Distribution of Patient and Disease Characteristics for Patients with Ovarian Masses

Patient characteristics	Statistical description of the Variables n (%)
Age	Mean+SD (years)
Malignant	52.10+9.02
Benign	42.33+8.82
Control	43.10+8.20
Histology	
Malignant	67
Serous Cystadenocarcinoma	54 (80.60)
Mucinous Cystadenocarcinoma	8 (11.94)
Dysgerminoma	2 (2.98)
Yolk sac tumor	1 (1.49)
Granulosa cell tumor	2 (2.98)
Benign	42
Serous Cystadenoma	16 (38.09)
Mucinous Cystadenoma	22 (52.38)
Mature Cystic Teratoma	2 (4.76)
Endometriotic Cyst	2 (4.76)
Control	26
Stage Wise Distribution	
Stage I	17 (25.37)
Stage II	24 (35.82)
Stage III	18 (26.86)
Stage IV	8 (11.94)

**Table 2:** Serum HE4 and CA-125 Levels for Patient Cohort According to Stage, Histology and Disease Status in Patients with Ovarian Masses

Variables	Serum HE4 (Mean±Std deviation) (pmol/l)	Serum CA-125 (Mean±Std deviation) (U/ml)
Disease status		
Malignant	96.76±38.96	303.61±336.61
Benign	49.33±10.00	22.76±17.37
Control	47.97±9.43	18.44±10.08
Stage wise		
Stage I-II	75.26±21.83	181.39±145.78
Stage III-IV	130.65±35.96	496.34±449.44
Histology		
Serous Cystadenocarcinoma	107.67±34.23	362.31±349.38
Mucinous Cystadenocarcinoma	51.76±26.44	48.65±17.81
Dysgerminoma	54.08±2.68	140.75±171.18
Yolk sac tumor	50.19±0.00	71.40±0.00
Granulosa Cell Tumor	48.17±6.10	17.50±7.77
Epithelial	100.45±38.15	321.84±342.53
Non-Epithelial	50.93±4.47	77.58±106

**Table 3:** Comparison of ROC-area under Curve, Sensitivity and Specificity of CA-125 and HE4 among Patients with Ovarian Masses

Variables	Serum HE4	Serum CA-125
Based on disease status (Malignant/Benign)		
Cut-off values based on ROC curve	69.8pmol/l	33.55 U/ml
Sensitivity at cut-off value	83.6	85.1
Specificity at cut-off value	100	90.48
Area under curve	0.899	0.939
95% Confidence interval	0.836-0.962	0.900-0.979
Positive predictive value^	100	93.44
Negative predictive value^ #^	86.08	79.17
Combined sensitivity	92.54	
Combined specificity	100	

PPV^, NPV#, Sensitivity/Specificity/PPV/NPV are expressed as %

**Table 4:** Diagnostic Value of Serum HE4 and CA-125 in Ovarian Cancer Staging and Histology

	Serum HE4	Serum CA-125
Diagnostic Value of Serum HE4 and CA-125 in Stage I/II		
Sensitivity	92.68	63.41
Specificity	100	90.48
Diagnostic Value of Serum HE4 and CA-125 in Stage III/IV		
Sensitivity	92.59	96.41
Specificity	100	90.48
Diagnostic Value of Serum HE4 and CA-125 in Epithelial Ovarian Cancer		
Sensitivity	91.94	88.71
Specificity	100	90.48
Diagnostic Value of Serum HE4 and CA-125 in Non-Epithelial Ovarian cancer		
Sensitivity	0	40
Specificity	100	90.48

Sensitivity and specificity are expressed as %

Studies have shown that HE4 may be more effective than CA-125 in early diagnosis of epithelial ovarian cancer (EOC) (30-31). Drapkin et al., (2005) and Montagnana et al. (2009) observed that the levels of HE4 were significantly increased in early ovarian cancer, while the levels of CA-125 did not increase and concluded that the release of HE4 occurred earlier than CA-125 in ovarian cancer patients. This was attributed to the lower molecular weight of HE4 protein when compared to the CA-125. The present data also showed that the diagnostic value of HE4 was superior to that of CA-125 in stage I and II patients (Table 4). HE4 was able to detect all the cases of early stage serous cystadenocarcinomas when compared to CA-125. However, for higher stages of serous adenocarcinoma, both the tumor markers had comparable diagnostic accuracy.

CA-125 is not very sensitive at detecting early-stage ovarian cancer and demonstrates lower specificity as CA-125 is often elevated in benign gynecological conditions (Meden and Meibodi, 1998; Ismail et al., 1994; Cheng et al., 2002; Kitawaki et al., 2005; Abrao et al., 1999). Hence, CA-125 has no established role in the early detection of gynecologic cancers. HE4 may be less frequently abnormal in benign gynecological conditions; therefore, HE4 combined with CA-125 may yield increased specificity in differentiating between benign and malignant conditions (Nolen et al., 2010; Hamed et al., 2013). The greatest benefit of highly specific differentiation between ovarian cancer and benign ovarian mass may well be found in the diagnosis of ovarian cancer at an early asymptomatic stage with good prognosis and survival benefits. 

The major advantage of HE4 lies in its specificity and improved detection of early stage ovarian cancers. Nolen et al., (2010) demonstrated that the sensitivity of the diagnosis of early ovarian cancers was improved from 74.2% to 91.7% by the combined detection of HE4 and CA-125. Hamed et al., (2013) also observed that with the combination of HE4 and CA-125, the sensitivity and PPV reached 96.7% and 97% respectively. It has been suggested that this combined detection was superior to the single detection by CA-125. The present study showed that the sensitivity and specificity were 92.54% and 100%, respectively, for the combined detection of HE4 and CA-125. This result was compared with HE4 used alone and it was observed that the sensitivity had increased. The results of present study revealed that the combined detection of HE4 and CA-125 contributed to the differential diagnosis of benign or malignant pelvic masses, but was not superior to the single detection of HE4 for the early diagnosis of epithelial ovarian cancer. 

Ovarian cancer subtypes have been shown to have distinct biomarker expression profiles. The diagnostic value of HE4 may vary with the histopathological type. The present study also observed that the level of serum HE4 was highest in serous adenocarcinoma patients and the difference compared with other types of ovarian cancer was statistically significant (p<0.01), although no statistically significant difference was observed among the mucinous adenocarcinoma and non-epithelial ovarian malignancies (Hogdall et al., 2007). Drapkin et al., (2005) observed that HE4 was not expressed in mucinous ovarian cancer and normal ovarian tissues; however, it was expressed in 50% of the ovarian clear cell carcinomas, 93% of the serous ovarian cancers and 100% of endometroid carcinomas of the ovary. 

Certain studies have investigated whether HE4 may be used as a marker to monitor disease progression and predict prognosis. Xu et al., (2010) in his study showed high preoperative blood levels of HE4 as a predictor of poor prognosis in patients with ovarian cancer. Present study also observed higher levels of HE4 and CA-125 in the advanced stage ovarian cancer patients which may signify adverse prognosis. 

Limitation of our study was small number of cases of non-epithelial ovarian cancer; hence applicability of this test cannot be generalized for all ovarian malignancies.

HE4 is a sensitive tumor marker for detecting epithelial ovarian cancer. HE4 was more sensitive then CA-125 in detecting the early stages of ovarian cancer. Main advantage of serum HE4 lies in its specificity to distinguish benign from malignant ovarian masses. HE4 improves the utility of CA-125 as a tumor marker in ovarian cancer, and the use of this combination might enable to improve detection of ovarian cancer patients.
